# Bioethics in popular science: evaluating the media impact of *The Immortal Llife of Henrietta Lacks* on the biobank debate

**DOI:** 10.1186/1472-6939-14-10

**Published:** 2013-02-28

**Authors:** Matthew C Nisbet, Declan Fahy

**Affiliations:** 1 School of Communication, American University, 4400 Massachusetts Avenue, NW, 20016-8017, Washington, DC, USA

## Abstract

**Background:**

The global expansion of biobanks has led to a range of bioethical concerns related to consent, privacy, control, ownership, and disclosure. As an opportunity to engage broader audiences on these concerns, bioethicists have welcomed the commercial success of Rebecca Skloot’s 2010 bestselling book *The Immortal Life of Henrietta Lacks*. To assess the impact of the book on discussion within the media and popular culture more generally, we systematically analyzed the ethics-related themes emphasized in reviews and articles about the book, and in interviews and profiles of Skloot.

**Methods:**

We conducted a content analysis of a population of relevant English-language articles and transcripts (n = 125) produced by news organizations and publications in the U.S., Canada, Great Britain/Ireland, and Australia/New Zealand. We scored each article for the emphasis and appearance of 9 ethics-related themes. These were informed consent, welfare of the vulnerable, compensation, scientific progress, control/access, accountability/oversight, privacy, public education, and advocacy.

**Results:**

The informed consent theme dominated media discussion, with almost 39.2 percent of articles/transcripts featuring the theme as a major focus and 44.8 percent emphasizing the theme as a minor focus. Other prominent themes and frames of reference focused on the welfare of the vulnerable (18.4 percent major emphasis; 36.0 percent minor emphasis), and donor compensation (19.2 percent major; 52.8 percent minor). Ethical themes that comprised a second tier of prominence included those of scientific progress, control/access, and accountability/oversight. The least prominent themes were privacy, public education, and advocacy.

**Conclusions:**

The book has been praised as an opportunity to elevate media discussion of bioethics, but such claims should be re-considered. The relatively narrow focus on informed consent in the media discussion generated by Skloot’s book may limit the ability of ethicists and advocates to elevate attention to donor control, compensation, patenting, privacy, and other ethical issues. Still, ethicists should view the book and a pending major TV film translation as opportunities to highlight through media outreach, consultation exercises and public forums a broader range of bioethical concerns that would otherwise be under-emphasized in news coverage. Such efforts, however, need to be carefully planned and evaluated.

## Background

More than $1 billion has been spent on establishing biobanks worldwide [1] and the sector’s commercial value is predicted to grow to more than $2.25 billion by 2015 [2]. At least 300 million human samples are stored in U.S. biobanks alone, and this number is estimated to be growing by at least 20 million a year. In the United Kingdom, a government-sponsored biobank launched in 2004 has already enrolled 500,000 donors, processing samples from 700 to 1,000 volunteers a day [3]. Yet as commercial repositories for tissues, fluids, genetic material, and correlated lifestyle data, biobanks raise a range of questions related to ethics, policy, and communication.

In a recent article at *Nature Biotechnology,* bioethicists and legal scholars note that “[a]lthough informed consent remains one of the most contested issues of biobank policy, other legal and ethical challenges also require careful attention” [4]. These include the protection of vulnerable subjects, the safeguarding of privacy, the communication to donors of research results, conflicts over patenting, access, and the need for open science, and the rights of donors to retain a property claim or control over their tissues. Experts more critical of biobank procedures and policies, such as U.S. bioethicist Lori Andrews, have argued the need for a tissue-rights movement in which members of the public become “conscientious objectors in the DNA draft,” a strategy intended to challenge status quo policy [5,6] p. 320. Negotiating all of these issues is complicated by a lack of clear national and international oversight [4].

In this study, we examine the media discussion generated by the 2010 best-selling popular science book *The Immortal Life of Henrietta Lacks* by U.S science writer Rebecca Skloot [5]. Many bioethicists and commentators have praised the book as a leading opportunity to constructively engage a broader public on the ethical issues related to biobanks and tissue research more generally. The book documents the story of Henrietta Lacks, a young African-American woman who died of cervical cancer at Baltimore, Maryland’s Johns Hopkins Hospital in 1951. Skloot described how cells removed from the body of the mother of five without her permission as she lay dying were given the label HeLa and, for a reason still unknown precisely to scientists, became the first human cell line to survive outside the body, contributing ultimately to vaccines, drugs and treatments for polio, hemophilia, HIV and several forms of cancer. Skloot weaves together three connected narratives: the story of Henrietta’s life; the story of how scientists used her cells to achieve extraordinary medical advances; and the story of how the Lacks family, particularly her daughter Deborah, struggled to come to terms with her mother’s legacy. Running through these stories is the history of exploitation of African-Americans for research by the medical establishment in the United States.

Most biobanks today collect finite amounts of tissue, very few of which are made into “immortal” cell lines as was the case with HeLa. In addition, many biobanks do not use tissues removed during medical procedures, as was the case with Lacks. Yet the story presented by Skloot had the potential to generate a broader discussion in the media and among the public about the ethical concerns related to biobanks and tissue research, a discussion that Skloot herself attempted to generate in media interviews, in the afterward to her book, and in prominent freelance articles.

### Biobanks as a communication challenge

In relation to biobanks and ethics, there are several major communication challenges. In order to effectively detect associations between genetic factors, environmental influences, and a specific disease, scientists require biobanks stocked with tens of thousands of samples. As a consequence, biobanks depend on participation from massive numbers of donors, who consent broadly to the use of their biological samples for a diversity of research purposes [7]. For example, in an effort to understand gene-environment interactions that influence disease across the U.S. population, the National Institutes of Health is considering a study that would involve 500,000 volunteers. Researchers would take biological samples and track subjects across a period of years and possibly decades. Without elevated attention and discussion of biobanks in the U.S., participation at the scale needed for the NIH proposed study is unlikely [8].

However, few members of the public have heard of biobanks, even though their own samples may already be stored or used by researchers. According to a survey analysis conducted in 2010, two-thirds of Europeans were unaware of biobanks, but those who had heard about the repositories were more likely to participate as donors and to give broad consent. The proportion of the public prepared to participate in biobanks varied by country and also depended in part on how much trust citizens in different countries placed in their respective governments. The authors of the European analysis suggest that increased media attention and debate can usefully contribute to greater knowledge and interest in biobanks. “Controversies don’t seem to lead people to reject the idea of biobank research per se,” they wrote. “Instead they facilitate the spread of information, and improve understanding and sharing of views on what is appropriate and acceptable use of samples.” As they conclude: “What is needed is a dialogue with the public, to explain the purposes of biobanks and how they operate, and to give people an opportunity to voice their concerns and conditions for their support and participation” [1].

Communication matters in other ways as well. A 2008 U.S. study employing 16 focus groups comprised of 141 subjects recruited from 6 different cities concluded that information and awareness are not only linked to a willingness to participate in biobank efforts examining gene-environment interactions, but also to a strong preference that the public be given ongoing choices on how research results could be accessed. In this case, public awareness not only appears to create demand for greater donor control and empowerment, but also creates ethical, logistical, and communication challenges specific to the effective and responsible release of research results [7].

Over the past decade, science organizations and institutions have increasingly turned to consultation exercises such as public meetings to facilitate public learning and dialogue about complex policy questions such as those posed by biobanks [9]. One notable study organized town meetings in 5 U.S. cities, consulting the public on biobank-related issues such as the protection of privacy, the sharing of research results, and the profits made by biotechnology companies [10]. Yet, as many previous studies suggest, even with increased investment in consultation exercises, the news and entertainment media remain the main forum through which the broadest and most diverse segments of the public are likely to learn about and form at least tentative opinions about complex biomedical topics. In this sense, consultation exercises provide the opportunity and means by which motivated members of the public can learn, discuss, and sort through ethical and legal issues related to biobanks. For much of the rest of the public, the news media and popular culture serve a surveillance function, calling their attention to an issue like tissue research, and in the process, defining its’ significance [11].

### The impact of a popular science best-seller

A major critical and commercial success, *The Immortal Life of Henrietta Lacks* appeared on the *New York Times* hardcover bestseller list for more than 11 months, and had sold, by 2011, more than 1.25 million copies in the U.S. alone. As of 5 August 2012, the book had been on the *New York Times* paperback non-fiction bestseller list for more than 18 months. The book is under development as an HBO film by producer Oprah Winfrey, and a young adult edition of the book is planned for readers ages 10 to 14 [12].

*The New York Times* wrote that the book is “from first page to last, a meditation on medical ethics – on the notion of informed consent, and on the issue of who owns human cells” [13]. *The Washington Post* said the book was an investigation of “a social wrong committed by the medical establishment” [14]. Britain’s *The Independent on Sunday* described the book as “a case study of race and medical ethics, of the problems that plague black society…” [15]. Novelist Hilary Mantel, writing in *The Guardian*, noted: “The dark, inhuman face of unpoliced science shows itself throughout this story, side by side with the bright face of discovery and humanitarian advance” [16]. The U.S National Academies awarded the book a 2011 Keck Prize for science communication, and cited Skloot’s work as: “A compelling and graceful use of narrative that illuminates the human and ethical issues of scientific research and medical advances” [17].

Best-selling science books do not merely engage and educate readers, but they also make policy-related arguments, shape news coverage, and become a topic of wider cultural discussion [18]. These books make complex scientific and ethical topics meaningful for audiences by presenting them in terms of specific themes and narratives. As organizing devices, narratives provide a specific temporal order of events, arranged in a dramatic plot, creating a rhetorical sequence which reaches a climax that leads to a resolution that helps audiences understand the moral lessons and conclusions to be taken from the story [19]. In other words, narratives make issues concrete and coherent, meaningful and memorable, emphasizing certain ethical themes and moral judgments over others [20]. Yet, the choice by journalists like Skloot to focus on some ethical considerations while paying less attention to others can have important implications for how readers understand a complex topic like tissue donation and research [21].

In interviews related to her book, Skloot provided insight on the narrative choices and specific themes that she used to structure her book. She described, for example, the importance of telling a personal story. As Skloot told Niemen Reports: “Among other things, my book is the history of tissue culture and the evolution of bioethics told through the story of a family.” The book’s narrative arc “was really the story of Deborah [Lacks]: her struggle to learn who her mother was, to come to terms with the cells,” said Skloot [22]. The commercial success and wide public interest in the book has been attributed strongly to the foregrounding of the Lacks’ story. As a review in *The Sunday Times* (UK) by Bryan Appleyard, noted: “The primary narrative concerns race, poverty – financial and educational – and the abyss that divides the scientific understanding of the human body from the people’s. It is her adoption of this primary narrative that makes Rebecca Skloot’s book such a gripping read” [23].

Yet by strongly foregrounding the individual case of Lacks, Skloot risked not only obscuring other important ethical issues but also limiting reader consideration of deeper questions related to biomedical research and the growing demand for biobanks. In comments, for example, Skloot noted that she intended the book to serve as a warning about the dangers of treating life instrumentally and of the need to think broadly about history, policy arrangements, and governance. As she told the *Wisconsin State Journal*: “It’s really important for scientists in general to think about the fact that there are human beings behind every biological sample that we use in a laboratory . . . Also, it's a story about how scientific progress happens faster than the regulations that govern it” [24]. She also told *The Calgary Herald* that the book relates to several current health policy issues, including access to health care for minorities, science education, and the importance of scientists communicating with non-specialists [25].

Skloot attempted to balance the personalized focus of the book’s main narrative with a 13-page afterward that reads like a more broadly contextualized policy brief. Yet even in this case, Skloot makes specific choices about how to define the relevant ethical issues involved in tissue donation and research. As she writes:

There are, essentially, two issues to deal with: consent and money. For most people, knowing if and how their tissues are being used in research is a far bigger issue than profiting from them. Yet when this book went to press, storing blood and tissues for research did not legally require informed consent, because the law governing such things doesn’t generally apply to tissue research [5] p. 317.

Her afterward and the specific themes emphasized mirror closely a 7,500 word feature that Skloot published at the *New York Times Magazine* in 2006. The feature, subtitled “The Tissue Industrial Complex,” is noteworthy for not mentioning the Henrietta Lacks case, but instead foregrounding at the opening of the article the growth in tissue storage and a corresponding lack of clear regulatory and governing arrangements. Yet in defining more broadly the problem, Lacks again leads readers back to informed consent as the solution. The apparent implication is that as long as informed consent is satisfied, then other questions such as donor control become secondary. Consider a leading example from the article, in which she discusses two major historic cases:

“The difference between Ted Slavin and John Moore wasn’t that Slavin owned his tissues and Moore didn’t . . . The difference was information. Someone told Slavin that his tissues were special and that scientists might want them. So he was able to control his tissues by establishing his terms before anything left his body. In other words, he was informed, and he gave consent. In the end, the question isn’t whether people have the ability to control their tissues; it is how much science should be obligated (ethically and legally) to put them in a position to do so” [26].

To date, even among academics, there has been limited in-depth analysis of the book’s substantive treatment of the ethical and policy questions related to tissue research, donation, and biobanks. An exception is an 18 June 2012 review essay that appeared at the conservative journal *The New Atlantis*. Writer Ari N. Schulman critiques efforts to allow the market to govern decisions about research, and warns that Skloot’s emphasis may lead others to view informed consent as a “panacea” for a range of unaddressed ethical problems [27].

In addition, there has yet to be a systematic analysis of how Skloot’s book has been discussed in media reviews, coverage, and commentary. Given the importance of the media to how the broader public understands the nature of bioethics and biobanks specifically, along with the special role that best-selling science books can play in reaching wider audiences, the purpose of this study is therefore to systematically evaluate the ethical themes emphasized in the media coverage generated by the book.

## Methods

In order to determine relevant reviews, news stories, features, commentaries, profiles, and interviews generated by the publication, promotion, and success of Rebecca Skloot’s *The Immortal Life of Henrietta Lacks*, we searched the LexisNexis and Factiva databases between January 1, 2009 and June 15, 2012, for English-language articles and transcripts containing in the full text the key words “Rebecca Skloot” or “Henrietta Lacks.” The time period searched covers both advance promotion, the release of the hardcover edition, the release of the paperback edition, and the related promotional efforts by Skloot and her publisher. We individually reviewed each of the initially identified articles and transcripts, discarding duplicates or non-relevant articles. We then compared our remaining articles and transcripts to those listed by her publisher at Rebecca Skloot’s promotional Web site, retrieving via the Web relevant articles or transcripts that were not included in the culled population.

This process resulted in a final population of 125 articles and transcripts. These included 52 book reviews, 43 news stories or features, 22 profiles or interviews of Skloot, and 8 editorials, columns, op-eds, or letters-to-the-editor. Of the print and Web articles, 88 appeared at newspapers, 9 at public affairs magazines such as *The Economist* or *Slate*, 7 at trade journals such as *Library Journal* or *Publishers Weekly*, 6 at science or medical journals such as *Nature* or *The Lancet*, 5 at news wire services such as the Associated Press or Reuters, 2 at science magazines such as *The New Scientist*, and 1 at the magazine *Entertainment Weekly*. Among the programs retrieved, 4 were transcripts of interviews with National Public Radio affiliated programs such *Science Friday* or *Fresh Air*, and 3 were interviews with TV network programs at ABC News and PBS.

To assess the ethical themes featured in the articles, we developed a coding typology (see Table 1) by first reviewing key texts or articles that identify the major principles of bioethics [28,29] as well as recent journal articles or reports specific to the ethical and policy implications of biobanks and tissue research [1,4]. We further refined the typology by then comparing our preliminary categories to the themes emphasized by Skloot in her book and by similar works intended for a popular audience such as Lori Andrews’ and Dorothy Nelkin’s *Body Bazaar: The Market for Human Tissue*[6]. We then evaluated the validity of the typology in a pilot study that informally reviewed the population of 125 articles and transcripts.

**Table 1:** Typology of ethical themes

**Theme**	**Description**
*Welfare of Vulnerable, Sanctity of Person* (Welfare)	Focus on possible mistreatment, exploitation of African Americans, other minority groups; low-income groups, veterans or soldiers, children, elderly, sick, and/or concerns over defining the body as an object, product, commodity, subject rather than a person.
*Informed Consent* (Consent)	Focus on notification/education of patient/donor. Discussion of lack of consent, or merits/weaknesses of different consent models such as specific consent, broad consent, or presumed/opt-out consent.
*Compensation, Benefit, Profit Sharing* (Compensation)	Focus on benefits to patient/donor including treatment, pay, or profit sharing; includes discussion of fairness and balance between financial benefits to patient and profits made by scientists and industry.
*Privacy, Discrimination, Sharing Results* (Privacy)	Focus on protecting privacy and/or use of information to discriminate, and/or conditions under which patient/donor should be notified of research result; ability of patient to express consent/desire to be contacted; possible harm or benefit to patient or donor.
*Patient Control, Data Access, Patenting* (Control)	Focus on patient/donor’s ability to control research applications or uses; i.e. ability to withdraw specimen from biobank or database; and/or researcher access and sharing of data; and/or discussion of patenting of specific therapies and diagnostic tools.
*Accountability, Regulation, Oversight* (Accountability/Oversight)	Focus on nature or absence of regulatory rules, oversight of public and private biobanks, management of tissue/DNA storage and use; and/or ability of patient/donor or third parties to seek regulatory or legal action.
*Scientific, Societal Progress* (Progress)	Focus on need for tissue/DNA donations and banking for scientific and social progress; emphasis on benefits of progress outweighing concerns over informed consent and other possible ethical issues.
*Public Education, Consultation* (Education)	Focus on need for government, experts, and institutions to educate the public about issues and/or focus on need for direct public consultation and public/stakeholder involvement in decision-making. Includes general reference to public opinion or beliefs.
*Advocacy, Activism* (Advocacy)	Focus on call for action for “tissue rights movement,” and direct advocacy on the part of public and social groups to challenge the status quo and to seek policies more in line with the public interest and/or to shift away from culture of commercialization etc.

Based on a previously developed coding methodology for analyzing coverage of biotechnology debates, for each article or transcript, we scored each theme as “not present = 0,” “present = 1,” or “outstanding focus/appearing in the lede/headline” of the article/transcript = 2 [30,31]. At the start of our coding process, we tested our inter-coder agreement on a sample of 40 of the articles and transcripts. Based on careful review and pilot testing, our eventual agreement on scoring decisions across themes was 80 percent or higher for the 40 articles scored in the sample. After establishing reliability, we then moved forward to individually code the rest of the articles and transcripts in the population.

## Results

As Table 2 indicates, 39.2 percent of articles and transcripts featured discussion of *informed consent* as the major ethical issue relevant to the Lacks case, or tissue research more generally, or both. Another 44.8 percent emphasized the theme as a secondary concern. In total, 84 percent of articles or transcripts mentioned *informed consent*. As an example, consider this lede from a review at the liberal *Mother Jones* magazine: “When Henrietta Lacks – a poor, African American tobacco farmer from Virginia – checked into Johns Hopkins Hospital with cervical cancer in 1951, she had no idea that tissue removed from her body without her consent would become one of the most important resources in medical history” [32].

**Table 2:** Percentage of articles featuring major/minor emphasis on ethical theme

***Theme***	***Consent***	***Welfare***	***Compensation***	***Progress***	***Control***
Major					
%	39.2	18.4	19.2	8.0	4.8
*N*	*49*	*23*	*24*	*10*	*6*
Minor					
%	44.8	36.0	52.8	26.4	18.4
*N*	*56*	*45*	*66*	*33*	*23*
Absent					
%	16.0	45.6	28.0	65.6	76.8
*N*	*20*	*57*	*35*	*82*	*96*
***Theme***	***Accountability***	***Privacy***	***Education***	***Advocacy***	
Major					
%	--	.08	1.6	0.8	
*N*	*--*	*1*	*2*	*1*	
Minor					
%	26.4	12.8	6.4	4.0	
*N*	*33*	*16*	*8*	*5*	
Absent					
%	73.6	86.4	92.0	95.2	
*N*	*92*	*108*	*115*	*119*	

Note: Analysis based on coding of population of 125 articles. Each theme or frame of reference was coded as “not present = 0,” “present = 1,” or “outstanding focus/appearing in the lede/headline” of the article/transcript = 2. Analysis of population of relevant articles, so all differences are significant. Some cells may not total 100 due to rounding.

Though the theme of *informed consent* dominated popular discussion of the book, also prominent was an emphasis on the *welfare of the vulnerable* (18.4 percent major emphasis; 36.0 percent minor emphasis) and *compensation* (19.2 percent major; 52.8 percent minor). In total, 54.4 percent of the articles or transcripts analyzed mentioned ethical considerations related to *welfare* and 72 percent mentioned considerations related to *compensation*. In discussing the theme of *welfare*, many articles/transcripts alluded to the unethical treatment of blacks historically by the medical establishment in the U.S. Specific to *compensation*, many reviewers or journalists noted - as Skloot does - the contrast between the profits generated by HeLa cells, and Lacks’ family inability to afford health insurance.

Ethical themes that comprised a second tier of prominence included *scientific progress*, *patient control*, and *accountability/oversight*. Thirty-four percent of articles and transcripts emphasized *scientific progress*, 23.2 percent emphasized *control*, and 26.4 percent emphasized *accountability*, though few featured these three themes as major considerations.

An example of the argument that scientific progress should be given greater weight than other ethical considerations appears in a news story run by the trade publication *Internal Medicine News*. In this case, a scientist is quoted as expressing concern over the attention that the book has generated: “Rebecca Skloot’s book on Henrietta Lacks ‘has raised sensitivity’ for many people about the potential ethical and confidentiality dangers of biomedical research ‘without a counterbalancing, responsible story being told about the benefits’ of this research…’” [33].

In emphasizing the theme of *control*, a review at the *Winnipeg Free Press* noted: “The courts have decided that once a bit of tissue is removed from our bodies, we lose all ownership rights and are not entitled to share in any profits derived. Even our own genes have been patented for private gain, preventing the development of cheaper and more effective methods to diagnose and treat disease as breast cancer" [34].

The theme of *accountability* is emphasized in an interview with Skloot published by the *Atlanta Journal Constitution*. When asked if an individual’s tissue today could be used without consent, Skloot replies: “There are certain laws that say you have to be informed when scientists do certain things. What happened with Henrietta’s family, a scientist coming to them just to do research, they would have to have permission for that. *But everything else is pretty loose*.” [emphasis added] [35].

Finally, far less prominent themes included *privacy*, *public education*, or *advocacy* with fewer than 15 percent of articles mentioning these important ethical considerations and very rarely as major points of emphasis. The theme of *privacy* appears, for example, in a report on the PBS program Religion & Ethics Newsweekly which focused on the issue of tissue donation, quoting bioethicist Jonathon Moreno: “If you take some of my tissues, my cells, and do genetic analysis, you’re learning not just about me, but in some measure, at least, you’re learning about my relatives. So we have to be very sensitive to the prospects of stigmatization” [36].

Education was addressed, for example, in a news story run by Reuters, though in cautionary terms: “As it is, Skloot said scientists fear logistical headaches if they start relinquishing control and giving out a lot of information to the general public. Patients may veto research projects involving their cells due to ethical qualms, for instance, or may demand revenues” [37]. The theme of advocacy and activism, meanwhile, is used in the closing paragraph of a review of the book in the *New York Times*: “The notion of ‘tissue rights’ has inspired a new category of activists. The question that comes up repeatedly is, if scientists or companies can commercialize a patient’s cells or tissues, doesn’t that patient, as provider of the raw material, deserve a say about it and maybe a share of any profits that result? Few people these days may be willing to take no for an answer” [38].

In Table 3, we examined the correlations among themes, an indicator of how themes might have been packaged together in discussion to offer a diagnostic (i.e. problem definition) and prescriptive (i.e. problem solution) account of the Lacks case or tissue research more generally. In this case, two distinct groupings of ethical themes appeared across articles. First, most prominently, discussion of *consent*, *welfare*, and *compensation* often appeared together in the same article, as indicated by their significant and positive correlations. Similarly, a focus on *welfare* and *control* also tended to appear together as themes in articles. This suggests that when describing the risks from biomedical research posed to vulnerable populations and individuals, that the themes of *consent*, *compensation*, and *control* tended to be discussed as remedies or courses of action.

**Table 3:** Correlations among ethical themes

	**(1)** **Cons**	**(2)** **Welf**	**(3)** **Comp**	**(4)** **Prog**	**(5)** **Cont**	**(6)** **Acct**	**(7)** **Priv**	**(8)** **Edu**	**(9)** **Adv**
(1) Consent	1.00								
(2) Welfare	.26**	1.00							
(3) Compensation	.26**	.15	1.00						
(4) Progress	−.01	.02	.11	1.0					
(5) Control	.19*	.20*	.05	.16#	1.00				
(6) Accountability	.16#	.00	.09	.14	.27**	1.00			
(7) Privacy	.11	.13	-.12	.06	.28**	.24**	1.00		
(8) Education	.05	-.09	-.01	.16#	.26**	.36**	.31**	1.00	
(9) Advocacy	.00	-.02	.14	.15	.23**	.29**	-.09**	.21*	1.00

Note: Analysis based on coding of population of 125 articles. Each theme or frame of reference was coded as “not present = 0,” “present = 1,” or “outstanding focus/appearing in the lede/headline” of the article/transcript = 2. For analysis of correlations among themes, “outstanding focus/appearing in lede/headline” and “present categories” were combined, leaving a dichotomous measure where “1 = theme present” and “0 = theme not present”.

Second, though discussed much less frequently, an alternative diagnostic and prescriptive framing of tissue donation and research also appeared in articles. Specifically, *accountability*, *education*, and *advocacy* all correlate significantly and in the positive direction. Similarly, *privacy* correlates with *accountability* and *education*, though not with *advocacy*. This suggests that when issues related to *accountability/oversight* or *privacy* were raised as ethical concerns, the need for either public *education* or *advocacy*, or both, were also likely offered as solutions or courses of action.

Finally, we examined differences in the prominence of ethical themes by article/transcript type. Reviews (28.8%), stories/features (12.5%), and opinion articles (14%) were more likely than profiles/interviews (4.5%) to feature a major/lede focus on the *welfare of the vulnerable* as an ethical theme. Opinion articles (37.5%) were more likely than other article types to feature a major/lede focus on considerations of *control*, an ethical dimension that was otherwise rarely emphasized.

## Conclusion

Our analysis indicates that media reviews, profiles, and stories reflected the major ethical themes emphasized by Skloot in her book and publicity efforts. These included an overwhelming focus on issues related to informed consent, the welfare of the vulnerable, the treatment of individuals as people rather than mere subjects, and considerations of compensation and profit sharing. Less frequently emphasized themes included discussion of the conditions under which the need for scientific progress should outweigh other ethical concerns, the control that donors might have over their specimens, the balance between patenting and access to research, and accountability or oversight relative to current policies and procedures. Rarely mentioned or emphasized were considerations related to donor privacy and access to research results, to public education, or to the role of advocacy and activism.

Relative to understanding the direct impact of Skloot’s book on public judgments, our findings should be interpreted cautiously. Our study identified the range of arguments and themes that were most readily available via the media for the public to draw upon in forming opinions and making decisions. These findings, however, should be paired with direct audience research employing surveys, focus groups, in depth interviews and other methods. In coding the results of open-ended surveys, however, the typology of themes that we developed can be usefully applied. The coding scheme can also be used in future research related to media coverage and policy discourse specific to biobanks, including the type of discourse that takes place at public meetings and events.

Given the nature of our study, it is also not possible to say with confidence if Skloot’s book was the principal driver of the focus on informed consent in reviews, profiles, news stories, and opinion articles. Journalists and reviewers are not only influenced by the content of the book (and related publicity materials), but also by broader cultural and political discourses about biomedical research. Yet regardless of the factors ultimately responsible for the strong emphasis on informed consent, the narrow focus may in fact limit the ability of ethicists and advocates to elevate attention to donor control, compensation, patenting, privacy, and other ethical issues. In this regard, even though some scientists have voiced concerns that Skloot’s book might disrupt current policy arrangements specific to tissue donation and research, our findings suggest that the book may in fact serve to reinforce them.

There are other important implications for the journalistic treatment and communication of bioethics to the broader public. First, in contrast to the emphasis that has been placed on public education to ensure broader participation and enrollment in biobanks, media discussion of the book involves little to no emphasis on a role for the public. This may be in line with a view of the book by journalists and reviewers as mainly a cultural commodity and entertainment product to be reviewed, rather than an important opportunity to discuss biomedicine more broadly. Skloot herself has been acclaimed as a model of marketing and self-promotion, and media discourse for the most part characterizes the public as spectators and consumers rather than as active participants in an emerging policy debate.

Similarly, because discussion of the book occurred largely in reviews, author interviews and profiles, the book has yet to serve as a catalyst for in depth news coverage of biobanks and related issues. Extended public radio interviews, however, did allow for richer discussions of bioethics than were featured in the arts and culture section of other news organizations. The greater attention in opinion articles to ethical themes related to control, access, and patenting suggests that opinion writers and editors more so than other journalists viewed it as their role to use Skloot’s book as an opportunity to raise a broader set of ethical questions. These types of discussions have the potential to contribute to public understanding of the complex ethical issues related to biobanking, especially as there is a lack of consensus on these questions, including the meaning of consent, among both the public and experts [39].

Yet despite this potential, the book has yet to prompt journalistic investigations of informed consent policies and practices, has resulted in very few backgrounders on the ethical and policy issues involved, and has generated little attention to the global growth of biobanks specifically. The best-seller has been hailed as an opportunity to elevate discussion of bioethics in the media more generally, but the failure so far to catalyze these types of stories – along with the narrow framing of ethical issues featured in coverage of the book – suggest that such claims should be re-considered.

Finally, the success of the book should demonstrate to bioethicists and scientists that there is an intense public appetite for compelling narratives about advances in medical research and the ethical issues involved. As has been done in some university reading initiatives, the book’s popularity can serve as the starting point for community dialogue and discussion of a broader and more diverse set of ethical themes and frames of reference. With a major HBO film expected, the film may further widen the scope of public interest and the opportunity for engagement. Such efforts, however, need to be carefully planned and evaluated. Ethicists, scientists, and others should view subsequent media manifestations of the book as opportunities to highlight bioethical issues that would otherwise be under-emphasized in news coverage, commentaries, and reviews.

## Competing interests

The authors declare that they have no competing interests.

## Authors’ contributions

MCN and DF contributed to the design of the study, the collection, analysis and interpretation of data, and the writing of the manuscript. MCN performed the statistical analysis. Both authors read and approved the final manuscript.

## Authors’ information

MCN is Associate Professor of Communication and Co-Director of the Center for Social Media at American University. His research investigates the role of communication in policymaking and public affairs, focusing on debates related to environmental science, emerging technologies, and biomedicine. DF is Assistant Professor at American University’s School of Communication, where his research centers on emerging methods and models of science journalism and the role of scientists in popular culture.

## Pre-publication history

The pre-publication history for this paper can be accessed here:

http://www.biomedcentral.com/1472-6939/14/10/prepub
